# Development of Genetic System to Inactivate a *Borrelia turicatae* Surface Protein Selectively Produced within the Salivary Glands of the Arthropod Vector

**DOI:** 10.1371/journal.pntd.0002514

**Published:** 2013-10-31

**Authors:** Job E. Lopez, Hannah K. Wilder, Reid Hargrove, Christopher P. Brooks, Karin E. Peterson, Paul A. Beare, Daniel E. Sturdevant, Vijayaraj Nagarajan, Sandra J. Raffel, Tom G. Schwan

**Affiliations:** 1 Department of Biological Sciences, Mississippi State University, Starkville, Mississippi, United States of America; 2 Laboratory of Persistent Viral Diseases, Rocky Mountain Laboratories, National Institute of Allergy and Infectious Diseases, National Institutes of Health, Hamilton, Montana, United States of America; 3 Laboratory of Intracellular Parasites, Rocky Mountain Laboratories, National Institute of Allergy and Infectious Diseases, National Institutes of Health, Hamilton, Montana, United States of America; 4 Research Technologies Branch, Rocky Mountain Laboratories, National Institute of Allergy and Infectious Diseases, National Institutes of Health, Hamilton, Montana, United States of America; 5 Bioinformatics and Computational Biosciences Branch, Office of Cyber Infrastructure and Computational Biology, National Institute of Allergy and Infectious Diseases, National Institute of Health, Bethesda, Maryland, United States of America; 6 Laboratory of Zoonotic Pathogens, Rocky Mountain Laboratories, National Institute of Allergy and Infectious Diseases, National Institutes of Health, Hamilton, Montana, United States of America; University of California, Davis, United States of America

## Abstract

**Background:**

*Borrelia turicatae*, an agent of tick-borne relapsing fever, is an example of a pathogen that can adapt to disparate conditions found when colonizing the mammalian host and arthropod vector. However, little is known about the genetic factors necessary during the tick-mammalian infectious cycle, therefore we developed a genetic system to transform this species of spirochete. We also identified a plasmid gene that was up-regulated *in vitro* when *B. turicatae* was grown in conditions mimicking the tick environment. This 40 kilodalton protein was predicted to be surface localized and designated the *Borrelia repeat protein A* (*brpA*) due to the redundancy of the amino acid motif Gln-Gly-Asn-Val-Glu.

**Methodology/Principal Findings:**

Quantitative reverse-transcriptase polymerase chain reaction using RNA from *B. turicatae* infected ticks and mice indicated differential regulation of *brpA* during the tick-mammalian infectious cycle. The surface localization was determined, and production of the protein within the salivary glands of the tick was demonstrated. We then applied a novel genetic system for *B. turicatae* to inactivate *brpA* and examined the role of the gene product for vector colonization and the ability to establish murine infection.

**Conclusions/Significance:**

These results demonstrate the complexity of protein production in a population of spirochetes within the tick. Additionally, the development of a genetic system is important for future studies to evaluate the requirement of specific *B. turicatae* genes for vector colonization and transmission.

## Author Summary

Relapsing fever spirochetes are a global yet neglected pathogen causing recurrent febrile episodes, nausea, vomiting, and pregnancy complications including miscarriage. Most species of tick-borne relapsing fever spirochetes are maintained in enzootic cycles, and given an approximately 20 year life span, the arthropod vector for *Borrelia turicatae* represents a reservoir for the pathogens. While *B. turicatae* has adapted mechanisms to efficiently colonize and survive within the vector, the genes necessary during the tick-mammalian infectious cycle are unknown. We have identified a gene that was designated the *Borrelia repeat protein A* (*brpA*). *brpA* was up-regulated in a portion of the spirochetes colonizing *Ornithodoros turicata*, the vector for *B. turicatae*. Developing a system to delete the gene in *B. turicatae* enabled the evaluation of the necessity of *brpA*. With the genetic system established for *B. turicatae*, a better understanding of the genetic constituents required during the tick-mammalian infectious cycle may be obtained.

## Introduction

Tick-borne pathogens must efficiently adapt to the arthropod vector and mammalian host to ensure survival of the microorganism. Species of relapsing fever spirochetes are such pathogens that colonize and are transmitted by argasid ticks. *Borrelia turicatae*, an agent of tick-borne relapsing fever distributed throughout the southern United States and Latin America, is maintained in enzootic cycles by *Ornithodoros turicata* [1]. After an infectious bloodmeal the spirochetes initially colonize the midgut, and given the short duration of the bloodmeal (5–60 min) [2], [3], salivary gland colonization is necessary for subsequent transmission. Furthermore, with efficient transovarial and transstadial transmission, the vector itself is considered the reservoir for the spirochetes [4].

To understand *B. turicatae* adaptation within the tick, identifying and characterizing the genes that are up-regulated during vector colonization is important. A microarray analysis identified a gene, which we designated the *Borrelia repeat protein A* (*brpA*), to be up-regulated when spirochetes were grown at 22°C, a temperature similar to the tick (unpublished results). Originally reported as BTA018 [5], BrpA was predicted to contain a signal peptide, suggesting the protein's surface localization. Given the significance of microbial surface proteins in pathogenesis, the BrpA was further investigated. The surface localization and production of the protein was determined, and a system to inactivate the *brpA* was developed to evaluate the necessity of the gene during the tick-mammalian infectious cycle. These results demonstrate the up-regulation of a *B. turicatae* gene within the vector, differential protein production during midgut and salivary gland colonization, and the ability to inactivate *B. turicatae* genes by allelic exchange.

## Materials and Methods

### Ethical statement

All work was performed in adherence to the United States Public Health Service Policy on Humane Care and Use of Laboratory Animals and the Guide for the Care and Use of Laboratory Animals. Murine studies were conducted in accordance with the Mississippi State University Institutional Animal Care and Use Committee, protocol # 11-091. Animal husbandry was provided in adherence to the United States Public Health Service Policy on Humane Care and Use of Laboratory Animals and the Guide for the Care and Use of Laboratory Animals. Rabbit serum was produced by Cocalico Biologicals, Inc and all animal protocols were approved by the Animal Care and Use Committee (animal assurance number A369-01).

### *B. turicatae* isolates, sequence, and *in silico* analysis of *brpA*

Isolates of *B. turicatae* used in this study were RML, TCB-1, TCB-2, 95PE-570, PE1-926, 91E135, and 99PE-1807 [6]. Production of recombinant BrpA, the inactivation of the gene, and tick transmission experiments were performed using the 91E135 isolate of *B. turicatae*. All *B. turicatae* isolates were grown in mBSK medium [7], [8].

The entire *brpA* locus in seven *B. turicatae* isolates was amplified using DNA sequences from *B. turicatae* 91E135 to design the primers *brpA* F flank and *brpA* R flank, which flank the gene (Table 1), and the internal sequencing primers *brpA* seq F1 and *brpA* seq R1 (Table 1). PCR amplification was performed with a DNA Engine Tetrad (Bio-Rad, Hercules, CA, USA) as previously described [9] using the Go*Taq* Flexi DNA Polymerase kit (Promega, Madison, WI, USA). Amplicons were visualized in an agarose gel containing GelRed (Phenix Research Products, Candler, NC, USA), and processed with the Qiagen PCR Purification kit (Qiagen Inc, Valencia, CA, USA). Sequencing reactions for *brpA*, were performed as previously described [6] and deposited to GenBank under sequence accession numbers KC859623-KC859629.

**Table 1:** Oligonucleotides and probes.

Primer	Sequence (5′->3′)
*brpA* F flank	CTTAAGTATCCCACAACAACTTAAAGCACA
*brpA* R flank	CACATTTCATTAAATATAATTTAAAGGTGGCAAAA
*brpA* seq F1	TTTAATATATAAGGAGAAGTGTAGTG
*brpA* seq F2	TAATGGTGAGAAAGCAGCCAGAG
*brpA* seq R1	GGCAAAATTTTTGTTTCGTCC
*brpA* seq R2	TCAATAAATTTCTCTAATTTAAGGTC
*brpA* F	AGGATTTGATCCAACAACCTCTTT
*brpA* R	ATCTTATTTTTTGTATGCGGCTGAT
*brpA* probe	6FAM-TGGTGACCAAAATATCTCTGAGGGATTACAGGAT-BBQ
*flaB* F	CCAGCATCATTAGCTGGATCAC
*flaB* R	GTTGTGCACCTTCCTGAGC
*flaB* probe	YAK-TGCAGGTGAAGGTGCGCAGGTT-BBQ
*brpA* F exp	CACCGTGAAAAAAATTAATTTATTAATTTTTTTAATTAGTA
*brpA* R exp	AGAACTAATTAAATGTCCAATGTCTTGTGACA
*flaB* F exp	ATGATCATAAATCATAATACGTCAGCTATAAATG
*flaB* R exp	TCTAAGCAATGATAATACATACTGAGGCAC
*brpA* F (−) 1000 bp	TATTTTCTTCATCAGGTTCTTCTTCATCAG
*brpA* R (+) 1000 bp	TAACGACAACGGTTTGGCAAAAATAATC
*brpA* F del-*NheI* A	T**GCTAGC**TAAATTGGGTGAATTTAGGTGTAATAATTTAAACTTTCTA
*brpA* R del-*AvrII* A	T**CCTAGG**TACACTTCTCCTTATATATTAAAATAATTTATAAATCAATATTCG
*flgB* promoter F-*AgeI* A	AT**ACCGGT**AGCACCCGGTAGCAAGTTAAAAAAATTTGAAATAAACTTG
*flgB* promoter R-*NdeI* A	ATT**CATATG**ACCCTCTATATCACAAAATACTTTTTACTATATTATA
*gent* R-*SpeI* A	AA**ACTAGT**CTCGGCTTGAACGAATTGTTAGG

A Bolded nucleotides indicate the restriction enzyme site.

*In silico* translation of *brpA* and amino acid alignments were performed using the MacVector 6.0 software package (Oxford Molecular, Oxford, UK). The algorithms used to predict the presence of a signal peptide, surface localization, and conserved motifs were the Basic Local Alignment Search Tool (BLAST) from NCBI, Motif Scan [10] on the MyHits web server, LipoP 1.0 [11], and ScanProsite [12]. The Vector NTI suite was used for amino acid alignments of BrpA [13].

### Southern blot analysis

Total genomic DNA was separated by reverse-field agarose gel electrophoresis and transferred to a MagnaGraph Nylon Transfer Membrane (Osmonics Inc., Minnetonka, MN, USA) as previously described [14], [15]. *brpA* seq F2 and *brpA* seq R2 (Table 1) were used to generate the probe for Southern blotting because the primers are within a conserved region of the gene. Hybridization probes were produced with the PCR DIG probe synthesis kit following the manufacturer's instructions (Roche Applied Science, Indianapolis, IN, USA). Probe amplification, hybridization, and development of the Southern blots were performed as previously described [6], [15].

### *O. turicata* colony

The ticks used in these studies originated from Kansas and were previously reared at the Rocky Mountain Laboratories for many years. We have fed adult *O. turicata* on naïve mice, performed immunofluorescent assays, and quantitative PCR to confirm that the ticks are uninfected. The ticks were housed in 15 or 50 ml ventilated tubes at 27°C and 85% relative humidity (RH) using a saturated solution of KCL [16].

An infected cohort of ticks was obtained by feeding second nymphal stage *O. turicata* on Swiss Webster mice that were needle inoculated intraperitoneally with 1×10^5^ wild type or mutant spirochetes. The following day spirochetes were detected in the blood and the ticks were allowed to engorge. Ticks were stored separately at 27°C and 85% RH.

### RNA isolation and analysis of *B. turicatae* in infected mouse blood, ticks, and spirochetes *in vitro* cultivated at 22°C and 35°C

RNA was extracted from three cohorts of three to five infected *O. turicata* ticks. The ticks were placed into 1.5 ml RNAse free centrifuge tubes (Qiagen), flash frozen using liquid nitrogen, and triturated into a powder using a pestle. Ground ticks were suspended in 100 µl of RNAlater RNA Stabilizing Reagent (Qiagen), centrifuged through the QIAshredder (Qiagen) column, and RNA was isolated using the RNeasy Mini kit (Qiagen) following the manufacturer's instructions including the on-column DNA digestion.

To obtain *B. turicatae* RNA from spirochetes grown *in vivo*, cohorts of five infected ticks were fed on Swiss Webster mice. Blood was obtained from the animals by tail nick, and spirochetes were visualized by dark field microscopy within four to five days after tick bite. Mice were sedated using 25 mg/ml Ketamine and 7.6 mg/ml Rompun at a dosage of 0.1 ml per 25 gm of body weight, and exsanguinated by intra-cardiac puncture. Whole blood was centrifuged at 5,000× g for 10 min at 35°C and the serum containing the spirochetes was removed and placed into a clean RNAse-free 1.5 ml centrifuge tube. *B. turicatae* was pelleted by centrifugation at 15,000× g for 20 min, supernatant was removed, and the spirochete-enriched pellet was suspended in 100 µl of RNAlater RNA Stabilizing Reagent (Qiagen), flash frozen using liquid nitrogen, and stored at −80°C until RNA was extracted. Spirochetes were centrifuged over a QIAshredder column (Qiagen) and RNA was isolated as described above.

*B. turicatae* RNA was isolated from low passage cultures (passage five) grown at 22°C and 35°C in mBSK medium. Spirochetes grown in 50 ml cultures were centrifuged at 8,000× g for 15 min, the pellets suspended in 100 ml of RNAprotect (Qiagen), flash frozen using liquid nitrogen, and stored at −80°C. Spirochetes were processed and RNA was isolated as described above.

RNA integrity was determined using the Agilent RNA 6000 Pico kit and the Agilent 2100 Bioanalyzer (Agilent Technologies, Waldbroon, Germany) following the manufacturer's instructions. RNA integrity values of 8.0 or higher were used for quantitative reverse transcriptase-PCR analysis (qRT-PCR).

### qRT-PCR

Duplex qRT-PCR was performed to determine the up-regulation of *brpA* in spirochetes grown at 22°C and within the tick compared to spirochetes grown at 35°C and in murine blood. Primer and probe sets (Table 1) for *brpA* (*brpA* F, *brpA* R, and *brpA* probe) and *B. turicatae* flagellin (*flaB* F, *flaB* R, and *flaB* probe) were used at a final concentration of 5 µM (probe) and 10 µM (primer) (TIB MOLBIL, LLC, Adelphia, NJ, USA). The reporter fluorophores for the *flaB* and *brpA* probes were YAK and 6-FAM, respectively, while the quencher was BBQ (Table 1). To determine similar genomic equivalents of *flaB* and *brpA*, duplex assays were performed with a DNA template (10 - 0.1 ng) isolated from *in vitro* grown spirochetes using the primer and probe sets stated above.

qRT-PCR assays were performed using the AgPath-ID™ One-Step RT-PCR reagents (Life Technologies) and the StepOne Plus ABI 96-well (Life Technologies) following the manufacturer's instructions. Assays were performed in triplicate using 10, 1, and 0.1 ng of RNA. To determine if DNA contaminated the RNA samples, qRT-PCR assays were also performed without the RT. *flaB* was used as the normalization gene [17], and differential expression was determined using the 2^−*ΔΔ*CT^ calculation.

### Expressing *B. turicatae brpA* and *flaB* as recombinant fusion proteins in *Escherichia coli*

Forward (*brpA* F exp) and reverse (*brpA* R exp) primers (Table 1) were used to amplify *brpA* using genomic DNA from *B. turicatae* 91E135. PCR amplification, cloning into the pET102/Directional TOPO vector, DNA sequencing, expression, and recombinant protein purification were performed as previously described [9]. *brpA* was expressed as a thioredoxin, 6×-His-labeled fusion protein.

*flaB* was expressed as a hemagglutinin fusion protein using a modified pEXP1-DEST expression vector (Invitrogen). Briefly, a Cm^R^-ccdB-attR2 fragment was amplified by PCR from pEXP1-DEST with Accuprime *pfx* DNA polymerase (Invitrogen) using primers pEXP1-HA-F and pEXP1-HA-R (Integrated DNA Technologies, Coralville, IA, USA), which incorporated a C-terminal sequence encoding the hemagglutinin (HA) epitope. The Cm^R^-ccbB-attR2-HA PCR product was cloned using the In-Fusion kit (BD Clontech, Mountain View, CA, USA) into *Eco*RI and *Hind*III-digested pEXP1-DEST to create pEXP1-HA-DEST. The cloning process resulted in the deletion of the *Hind*III restriction site in pEXP1- DEST and generation of a new *Hind*III restriction site 5′ of the HA-tag sequence. *flaB* was amplified by PCR from *B. turicatae* genomic DNA with Accuprime *pfx* DNA polymerase using primers *flaB* F exp and *flaB* R exp (Table 1). The amplicon was cloned using the In-Fusion kit into *Bsr*GI and *Hind*III-digested pEXP1-HA-DEST to create pEXP1-HA::*flaB*.

### Producing antisera to recombinant BrpA (rBrpA) and recombinant FlaB (rFlaB)

Rabbit anti-rBrpA or chicken anti-rFlaB immune serum was produced by Cocalico Biologicals, INC. Preimmunization serum samples were collected, and two rabbits and two chickens were immunized twice intraperitoneally with 50 µg of rBrpA and rFlaB, respectively, using complete Freund's adjuvant. Three subsequent immunizations were performed at two week intervals using incomplete Freund's adjuvant. Serum samples were collected and tested for specificity to rBrpA and rFlaB by immunoblotting.

### SDS-PAGE and immunoblotting

All SDS-PAGE and immunoblot assays were performed using Any kD Mini-PROTEAN TGX precast gels (BioRad, Hercules, CA). Proteins were transferred at 100 volts for 1 hr onto polyvinylidene fluoride (PVDF) membranes with the Mini Trans-Blot Cell (BioRad) and antibody binding was detected with the ECL Western blotting reagent (VWR, Atlanta, GA, USA).

To determine the production of rBrpA, uninduced and induced *E. coli* BL21 Star (DE3) cells (Life Technologies) were electrophoresed and transferred to PVDF membranes following the manufacturer's instructions. PVDF membranes were probed with anti-poly-histidine peroxidase conjugate (Sigma-Aldrich, St. Louis, MO, USA) at a dilution of 1∶4,000. The immunogenicity rBrpA was evaluated using serum samples at a 1∶200 dilution from mice infected by tick bite.

One microgram of rBrpA and protein lysates from wild type and *ΔbrpA* spirochetes grown at 22°C and 35°C were prepared for immunoblotting as previously described [18], electrophoresed, and transferred to PVDF membranes. Immunoblots were probed with rabbit and chicken serum samples generated against rBrpA and rFlaB, respectively, at a 1∶200 dilution. To determine the antigenicity of BrpA, immunoblots were also probed with serum from mice infected by tick bite. HRP-rec-protein G (Life Technologies) was used as a secondary molecule when immunoblots were probed with rabbit serum, while goat anti-chicken IgY-HRP (Life Technologies) was used to detect FlaB binding.

### Cryosectioning and immunofluorescent laser scanning confocal microscopy (IF-LSCM)

IF-LSCM was performed on *B. turicatae* grown at 22°C, in infected murine blood, and ticks. 1×10^8^ *in vitro* grown spirochetes were centrifuged at 10,000× g for 5 min, the pellet washed with 1× PBS MgCl_2_, and suspended in 500 µl of 1× PBS MgCl_2_. Thick smears were produced on microscope slides using 10–50 µl suspension of spirochetes, air dried, and fixed in methanol for 30 min. Production of FlaB and BrpA was determined as described below.

Thin smears were produced from infected blood obtained from mice five days after cohorts of *B. turicatae*-infected ticks had fed on the animals and spirochetes were visible. Infected *O. turicata* were cryopreserved at −60°C and 20 µm longitudinal sections were cut using the Lecia CM1850 cryostat (Lecia Microsystems Inc, Buffalo Grove, IL, USA). Thin smears and tick sections were fixed in methanol and acetone for 30 min, respectively, air dried, and incubated in 1× PBS MgCl_2_ containing 0.75% bovine serum albumin (BSA) for 30 min.

FlaB production was assessed by probing thick smears of cultured bacteria, thin smears of infected blood, and cryosectioned ticks with chicken anti-rFlaB (positive control) diluted 1∶20 followed by Alexa Fluor 568 goat anti-chicken IgY (Life Technologies) diluted 1∶200. To detect BrpA, rabbit anti-rBrpA was diluted 1∶20 followed by Alexa Fluor 488 goat anti-rabbit IgG diluted 1∶200. IF-LSCM was performed with a Zeiss Axiovert 200M microscope (Carl Zeiss Microscopy, Munich, Germany), and images in the red and green channels were assembled using IMARIS imaging software (Andor Technology, Belfast, Ireland).

### Proteinase K treatment of *B. turicatae*

*B. turicatae* was grown at 22°C to 1×10^8^ spirochetes per ml and treated with proteinase K as previously described [19]. *B. turicatae* was harvested after 30, 60, 90, and 120 min and a final concentration of 5 mg/ml Phenylmethylsulfonyl fluoride in isopropanol was added to the spirochetes to inhibit proteinase K. Samples were electrophoresed, transferred to PVDF membranes, and membranes were probed with rabbit anti-rBrpA or chicken anti-rFlaB serum as described above. Assays were performed three times.

### Developing a construct for gentamycin resistance

The nucleotide sequence for the *Borrelia hermsii* flagellar basal body rod protein (*flgB*) promoter [8] was used to identify homologous sequence in *B. turicatae*. The *B. turicatae flgB* promoter was amplified with primers *flgB* promoter F and *flgB* promoter R, *Age*I and *Nde*I sites at the 5′ and 3′ end of the amplicon, respectively. The *B. turicatae flgB* promoter was ligated to the 5′ end of the gentamycin acetyl transferase gene (NCBI accession #: U22104) after digesting the pBhSV-2::*flgB gent* [8] with *Age*I and *Nde*I, replacing the *B. hermsii flgB* promoter with the *B. turicatae* promoter, forming pBhSV-2::*Bt* P*_flgB_-gent*.

### Constructing the suicide vector

Primers *brpA* F (−) 1000 bp and *brpA* R (+) 1000 bp (Table 1) and the Expand high-fidelity polymerase (Roche, Indianapolis, IN) were used to amplify *brpA* including 1000 base pairs up- and down-stream of the gene. The amplicon was cloned into the pCR-XL-TOPO plasmid (Life Technologies) and transformed into Top10 *E. coli* (Life Technologies). *NheI* and *AvrII* restriction sites were added onto the 5′ end of primers *brpA* F del and *brpA* R del (Table 1), respectively, and *brpA* was removed from the pCR-XL-TOPO by PCR amplification. P*_flgB_*_-_gent was amplified from pBhSV-2::*Bt* P*_flgB_-gent* using *flgB* promoter F and *gent* R *Spe*I primers (Table 1), adding *Avr*II and *Spe*I restriction sites, and cloned into the pCR-XL-TOPO containing *brpA* flanking DNA, forming the deletion construct.

### Transformation of *B. turicatae*

*B. turicatae* 91E135, passed seven times in mBSK medium at 35°C, was transferred into 500 ml of fresh mBSK and competent cells were made as previously described for *B. hermsii* [8]. Spirochetes were electroporated in 0.2 cm cuvettes (BioRad) and transferred into 5 ml of mBSK medium for 24 hr at 35°C. The 5 ml cultures were placed into 40 ml of mBSK containing a final concentration of 40 µg/ml of gentamycin. Within 5 days live spirochetes were visualized in the 45 ml cultures and 1 ml was transferred into 4 ml of fresh mBSK medium containing gentamycin. Once the bacteria attained a density of 1×10^8^ spirochetes/ml, the transformants were cloned in 96-well flat-bottom plates by limiting dilution from 1×10^4^ - 1×10^−3^ spirochetes/ml, and clonality was determined by Poisson statistics. PCR was performed to confirm the deletion of *brpA*, while immunoblotting was performed on protein lysates from *ΔbrpA* mutants grown at 22°C to confirm the protein was no longer produced. For PCR analysis, genomic DNA was extracted with the Wizard Genomic DNA Purification kit (Promega, Madison, WI, USA) and PCR was performed as described above. For SDS-PAGE and immunoblotting, protein lysates were prepared as previously described [18], and assays were performed as described above.

### Tick transmission studies and quantitative PCR (qPCR)

Cohorts of second stage *O. turicata* nymphs were infected with wild type or mutant *B. turicatae* by feeding the ticks on spirochetemic mice that were previously inoculated with 1×10^5^ bacteria. For subsequent transmission studies, cohorts of five ticks infected with wild type spirochetes or *ΔbrpA* mutants were allowed to feed on groups of five mice sedated with 25 mg/ml Ketamine and 7.6 gm/ml Rompun at a dosage of 0.1 ml per 25 gm of body weight. To evaluate transstadial transmission, the studies were repeated with the same cohort of ticks after they molted. In both studies, 2.5 µl of blood was collected daily by tail nick for 12 consecutive days, placed into 47.5 µl of Sidestep Lysis and Stabilization Buffer (Agilent Technologies), and stored at −80°C.

qPCR on infected blood was performed as previously described [20]. Primer and probe sets (TIB MOLBIOL, LLC) were designed for *B. turicatae flaB* (Table 1). All samples were assayed in triplicate, and a standard curve was developed using *in vitro* grown spirochetes at concentrations of 1×10^2^ to 1×10^8^ bacteria/ml. qPCR assays were performed with the 96-well ABI Step-One Plus Instrument (Applied Biosystems) in 20 µl reactions containing 2× Brilliant qPCR Master Mix (Agilent Technologies). Standard curves were constructed by plotting CT-values and spirochete concentrations, and the equation for the best-fit line was calculated using Microsoft Excel. R^2^ values for the best-fit line were ≥0.98.

## Results

### Plasmid mapping and amino acid sequence analysis of *brpA* from *B. turicatae* isolates

*brpA* was first identified by a microarray analysis to be up-regulated in spirochetes grown at 22°C, a growth temperature similar to the tick environment (data not shown). Searching the *B. turicatae* chromosome (GenBank accession number CP000048) and partially assembled plasmid sequences indicated that *brpA* was plasmid-encoded [5], and Southern blotting further mapped *brpA* to the large linear plasmid (data not shown).

**Figure 1:**
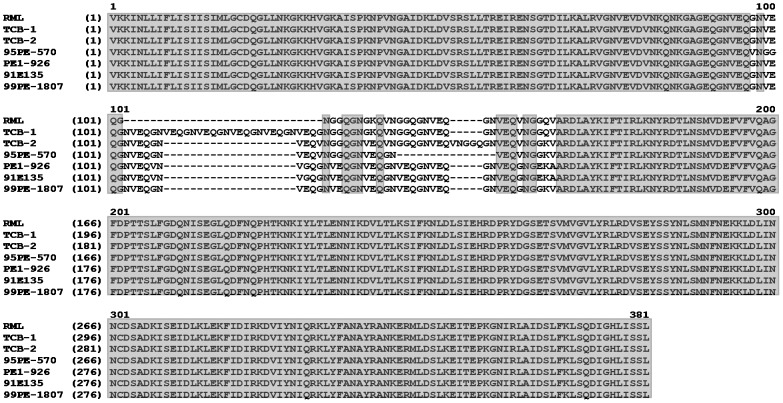
Amino acid alignments of BrpA from seven isolates of *B. turicatae*. Shaded grey are conserved amino acids, with the unshaded region depicting the Gln-Gly-Asn-Val-Glu repeats.

Amplifying the *brpA* locus from the seven isolates of *B. turicatae* produced a product of the expected size, and the sequenced amplicons confirmed the presence of the gene in all isolates. The amino acid sequence alignment indicated high sequence identity at the amino and carboxy terminus of the protein (Figure 1). Interestingly, the alignments also revealed a redundancy in the amino acid sequence Gln-Gly-Asn-Val-Glu, with TCB-1 displaying the greatest number of repeats (Figure 1).

### *In silico* analysis of BrpA

Sequence analysis of the *brpA* locus indicated the gene was not associated with an operon and was predicted to be 40 kilodaltons (data not shown). BLAST analysis of BrpA identified that the NxE motif within Gln-Gly-Asn-Val-Glu was similar to putative calcium binding proteins and the *Plasmodium* myosin-like proteins. The motif also shared similarity to the *Anaplasma marginale* Major surface protein 1 (Msp 1) [21]. BrpA was predicted to contain a signal peptide and lipoprotein motif, suggesting the protein was surface localized.

### *brpA* is up-regulated at 22°C and within infected ticks

Prior to determining differential *brpA* expression *in vitro* and *in vivo*, a series of assays evaluated the efficacy of *flaB* as the normalizing gene. *flaB* has been used for expression normalization in *B. hermsii* [17], and we confirmed similar transcript levels of *B. turicatae flaB* using RNA from spirochetes grown at 22°C and 35°C (Figure S1A). Performing the assays without the RT enzyme validated that the RNA samples were not contaminated with DNA (data not shown). Consequently, we assumed that *flaB* was equally expressed by *B. turicatae* during tick and mammalian infection. Moreover, using *B. turicatae* genomic DNA in the assays confirmed comparable C_T_ values for *flaB* and *brpA*, indicating similar genomic equivalents for these genes (Figure S1B).

qRT-PCR using RNA isolated from *B. turicatae* grown *in vitro* at 22°C and 35°C, in infected murine blood and infected ticks indicated that *brpA* was up-regulated at the lower temperature and in ticks (Table 2). Performing the assays without the RT enzyme demonstrated that the RNA preparations were free of DNA contamination (data not shown).

**Table 2:** Differential regulation of *brpA*.

Gene	Fold change at 22°C vs 35°C	Fold change within the tick vs blood
*flaB*	1.1	UD^A^
*brpA*	28.0	5.3

A Undetermined.

### Immunogenicity, production, and surface localization of BrpA during *B. turicatae* cultivation at 22°C

**Figure 2:**
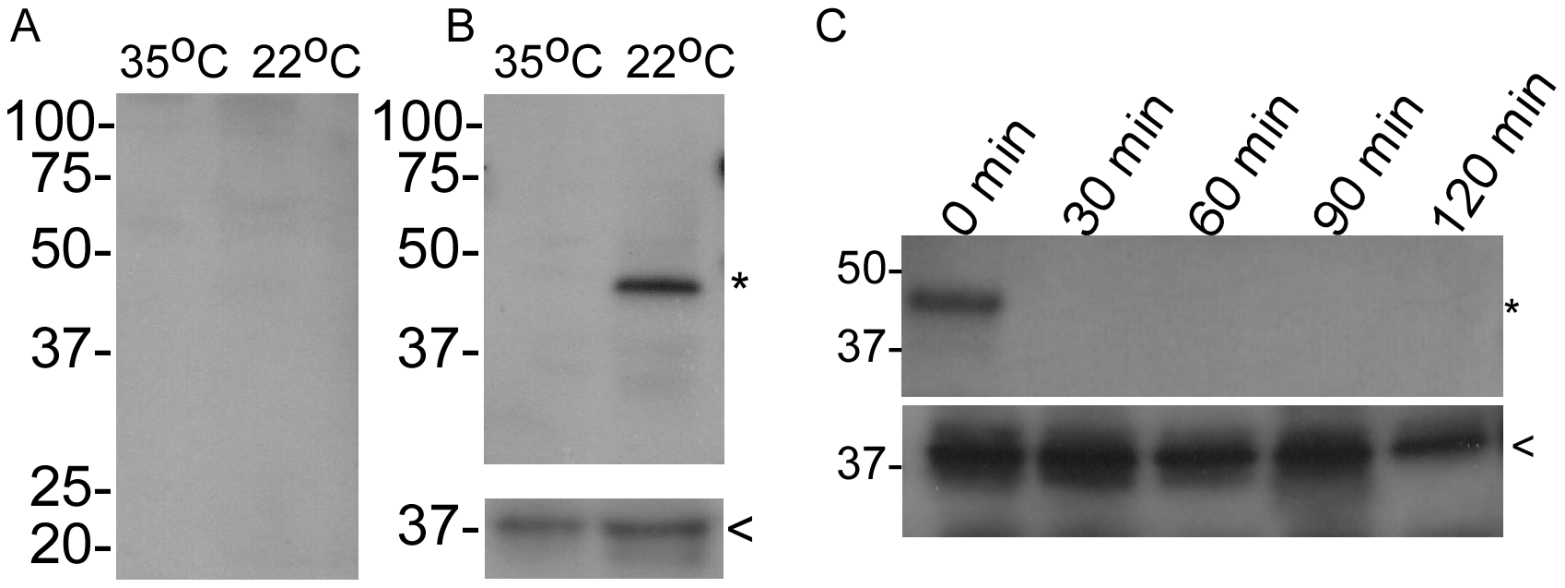
Temperature mediated production and surface localization of BrpA. A rabbit pre-immunization serum sample was used as a negative control (A), while hyperimmune serum generated against rBrpA was used to determine differential protein production at 22°C and 35°C (B). Immunoblots were also probed with chicken serum generated against *B. turicatae* rFlaB (B lower panel). Surface localization assays were performed by immunoblotting using the rabbit serum generated against rBrpA (C upper panel) and chicken serum generated against rFlaB (C lower panel). Molecular mass is depicted in kilodaltons on the left of each blot. The asterisk and arrowhead indicate the expected size of BrpA and FlaB, respectively.

Immunoblot analysis using serum samples from mice infected by tick bite indicated that rBrpA was not immunogenic (data not shown). Moreover, immunoblotting using serum samples from rabbits immunized with rBrpA demonstrated up-regulation of the native protein when spirochetes were grown at 22°C when compared to 35°C (Figure 2 A and B). Reactivity against FlaB with the samples indicated that similar amounts of spirochete lysates were electrophoresed (Figure 2B insert). The susceptibility of BrpA to digestion with proteinase K suggested that the protein was localized on the outer surface of the spirochetes (Figure 2 C). Detecting the periplasmic protein, FlaB, verified that the outer membrane of *B. turicatae* remained intact during the proteinase K treatments (Figure 2 C lower insert).

**Figure 3:**
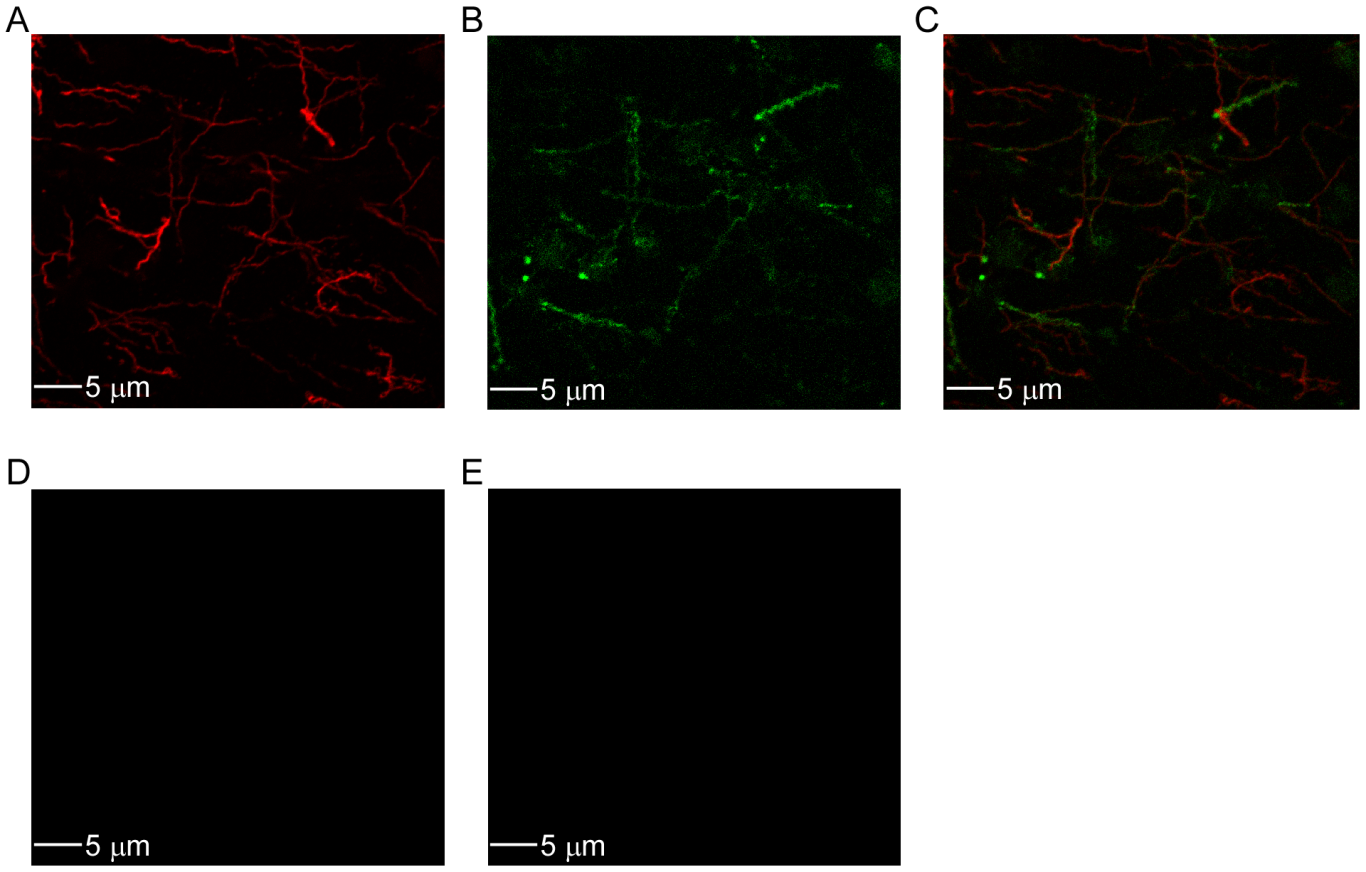
IF-LSCM of spirochetes cultivated at 22°C. Chicken and rabbit serum generated against rFlaB (A) and rBrpA (B), respectively, were used to determine protein production within a population of spirochetes. Images were overlaid and spirochetes producing BrpA were counted (C). Chicken (D) and rabbit (E) preimmunization serum samples were used as negative controls. The secondary antibodies used were Alexa Fluor anti-chicken IgY 568 and anti-rabbit IgG 488. 5 µm bars are shown in each panel.

Performing IF-LSCM using rabbit and chicken serum generated against rBrpA and rFlaB, respectively, demonstrated that BrpA was present in a portion (24.6%) of the spirochete population during cultivation at 22°C (Figure 3 A–C). Rabbit and chicken pre-immunization serum samples were not reactive to BrpA and FlaB, respectively (Figure 3 D and E). Collectively, these results further suggested that BrpA is a tick-associated surface protein.

### Production of BrpA by spirochetes in infected blood and ticks

**Figure 4:**
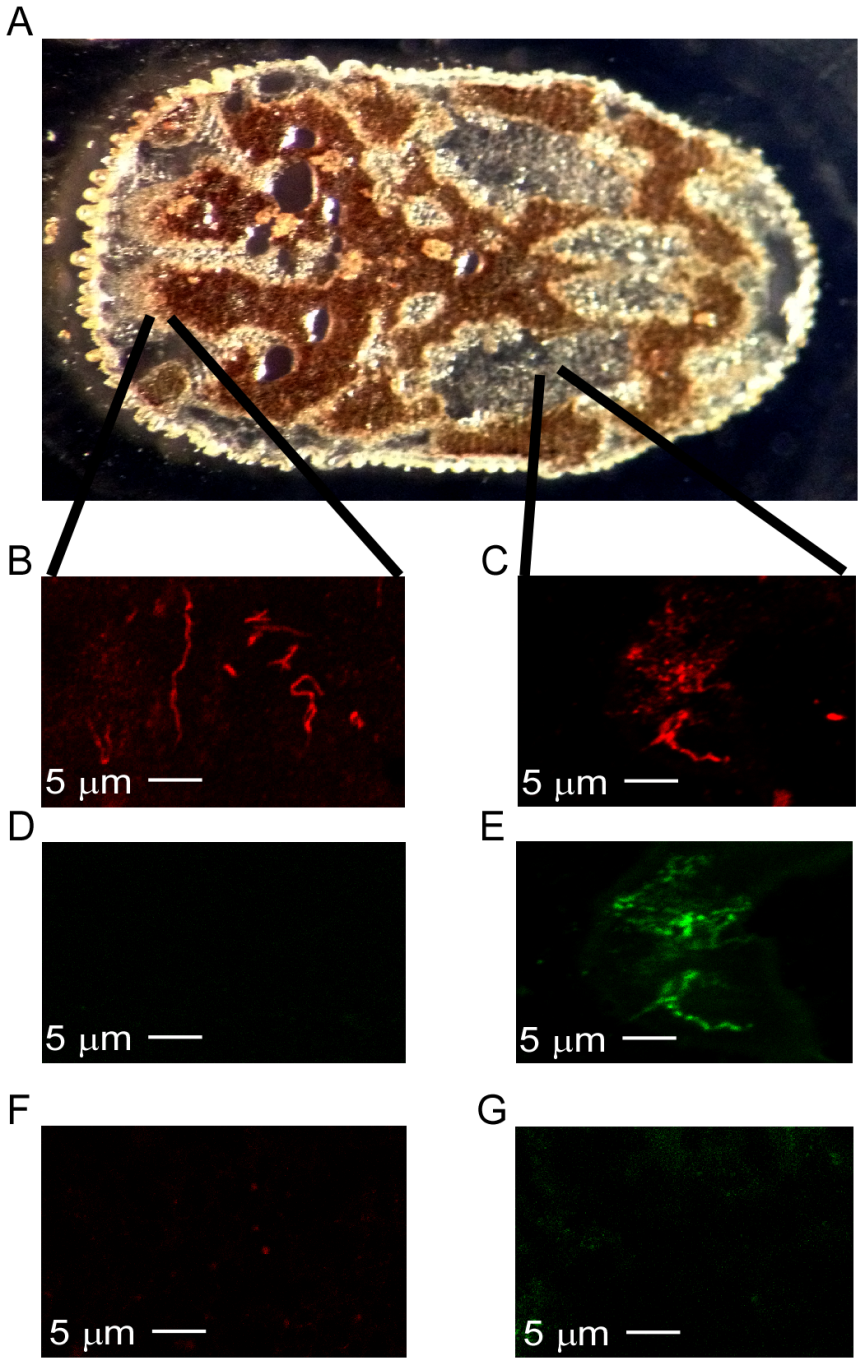
IF-LSCM of cryosectioned ticks. 20 µm longitudinal cryosections (A) were double labeled for FlaB (B and C) and BrpA (D and E) using chicken and rabbit serum samples generated against each recombinant protein. Pre-immunization serum samples were used as negative controls on infected ticks (F and G). The secondary antibodies used were Alexa Fluor anti-chicken IgY 568 and anti-rabbit IgG 488. 5 µm bars are shown in each panel.

Cryopreserving, sectioning (Figure 4 A), and performing IF-LSCM on infected *O. turicata* indicated increased BrpA production by *B. turicatae* in the tick salivary glands compared to the midgut. Probing tick sections with anti-rFlaB serum followed by the secondary antibody visualized the total spirochete population in the midgut (Figure 4 B) and salivary glands (Figure 4 C). Interestingly, BrpA production in the midgut (Figure 4 D) was diminished when compared to the protein's production within the salivary glands (Figure 4 E). IF-LSCM was also performed with chicken and rabbit preimmunization sera as a control for nonspecific binding (Figure 4 F and G). Counting spirochetes in three thin smears of infected murine blood, the midgut, and salivary glands from three ticks indicated that 29.3% of the spirochetes within the salivary glands produced BrpA compared to only 0.67% of the spirochetes in the midgut, and 10.0% in infected blood.

### Inactivation of *B. turicatae brpA*

**Figure 5:**
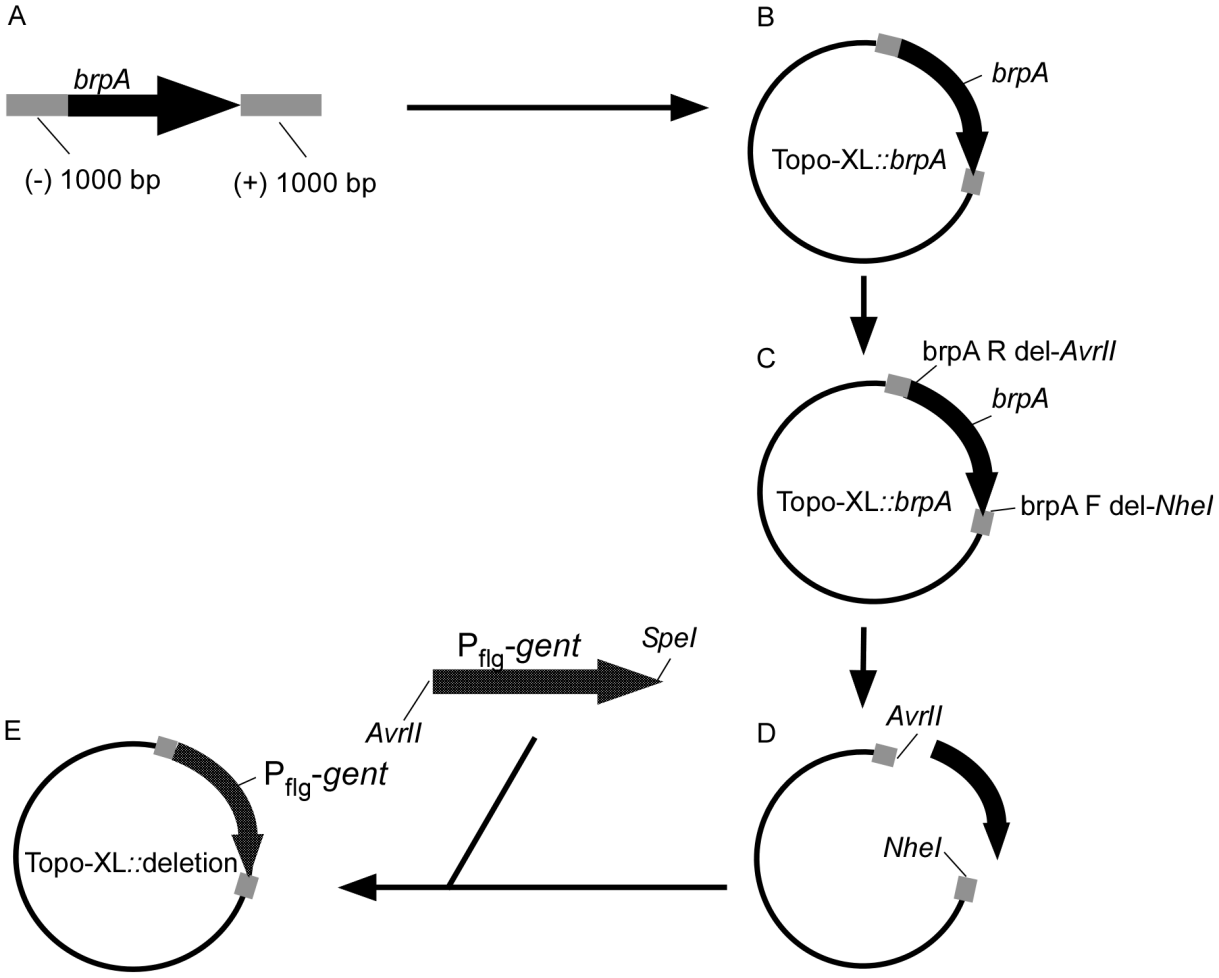
Constructing a deletion vector for *brpA.* * brpA* was amplified including 1,000 nucleotides up and down stream of the gene (A) and cloned into the TopoXL vector, constructing Topo-XL::*brpA* (B). *brpA* was removed from the vector by PCR amplification adding *AvrII* and *NheI* restriction sites (C and D) and the amplicon was double digested. The *B. turicatae flgB_P_-gent* was amplified adding *AvrII* and *SpeI* restriction sites, double digested, and the knockout vector constructed by ligation (E).

**Figure 6:**
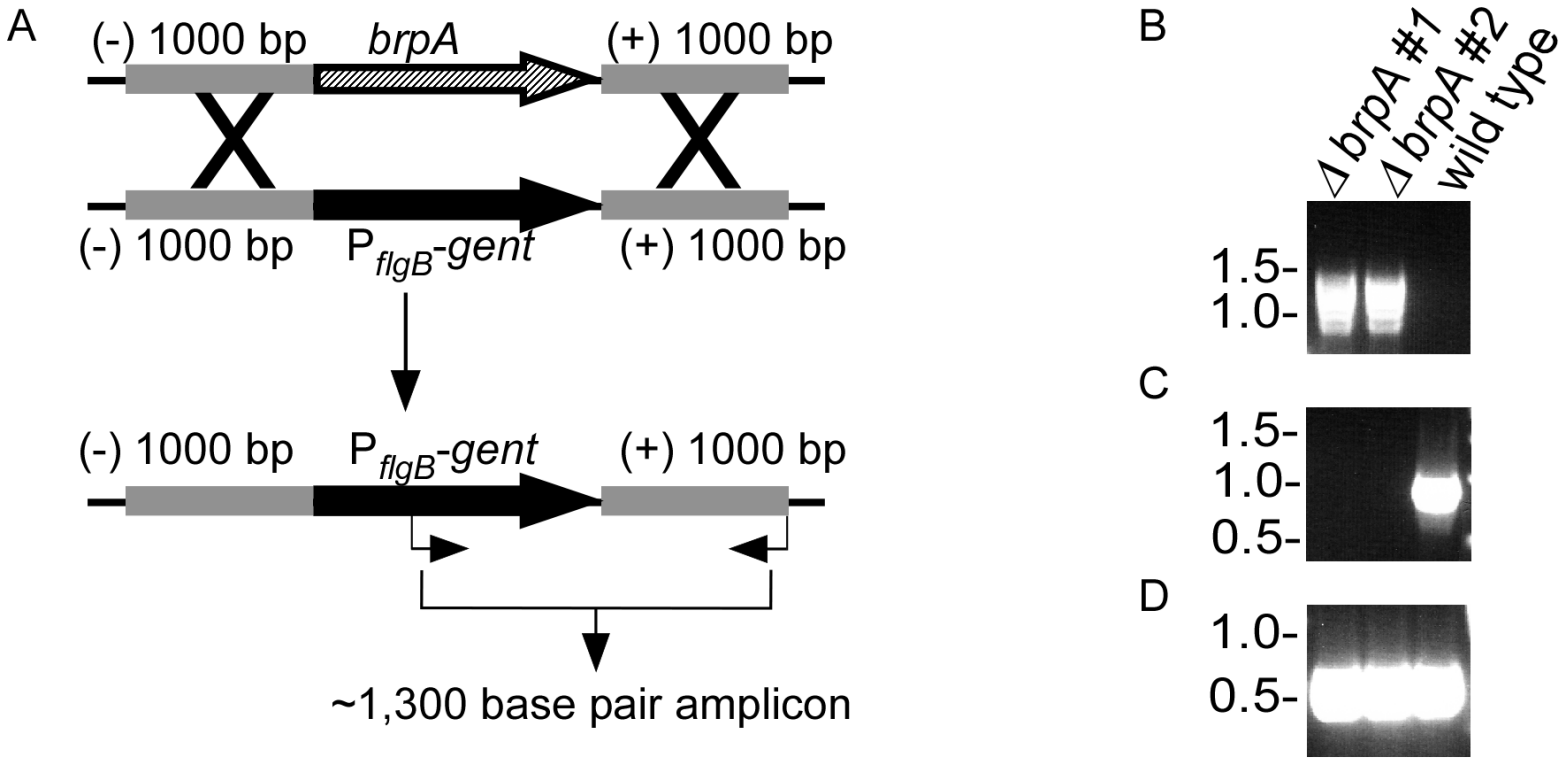
Inactivation of *brpA* by allelic exchange. The gentamicin acetyl transferase gene was flanked by 1,000- and down-stream of *brpA* forming P*_flgB_-gent*, and *brpA* was displaced by allelic exchange (A). Deletion of *brpA* was confirmed by PCR analysis using primers located within *gent* and 1,000 bp downstream of the gene (A and B), primers within *brpA* (C), and *flaB* (D). Arrow heads (A) indicate the primer location and the expected amplicon size. Molecular masses are shown at the left of the gel.

**Figure 7:**
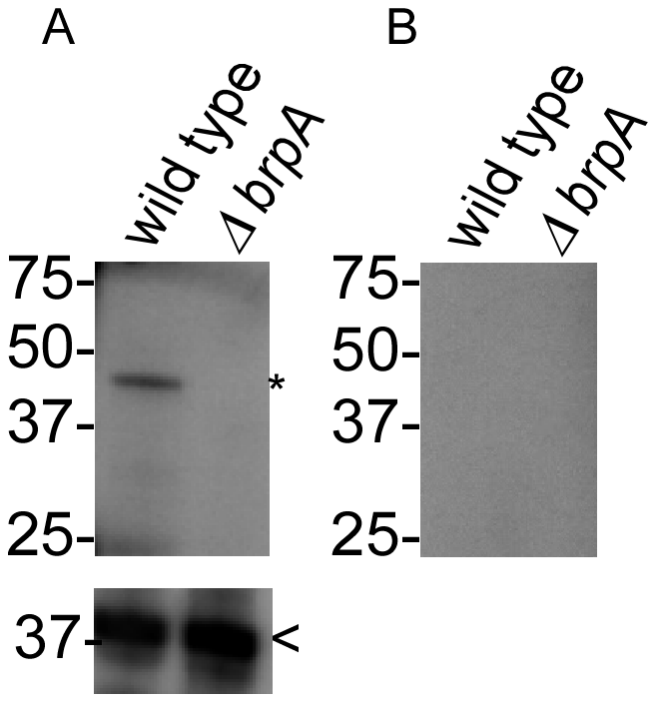
Loss of BrpA production in *ΔbrpA* mutants. Wild type and *ΔbrpA* mutants were grown at 22°C and immunoblotting performed using rabbit serum generated against the recombinant protein (A) and pre-immunization rabbit serum (B). The asterisk and arrowhead indicate the expected size of BrpA and FlaB, respectively.

Given the differential expression of *B. turicatae brpA* during the tick-mammalian infectious cycle, a genetic system was developed to assess the necessity of this gene for tick colonization and establishing murine infection. *brpA* deletion constructs were developed using the Topo-XL vector (Figure 5 A–E). Sequencing the deletion vector confirmed that the 1,000 base pairs up- and down-stream of P*_flg_-gent* were in frame and identical to the genomic DNA sequence (data not shown), and *brpA* was inactivated by allelic exchange (Figure 6 A–D). Transforming *B. turicatae* demonstrated the ability to genetically manipulate low passage spirochetes, and two clones were produced by limiting dilution, *ΔbrpA #1* and *ΔbrpA #2*. PCR analysis using primers localized within the gentamicin acetyl transferase gene and the 1,000 base pair region downstream of the gene produced a 1,300 base pair amplicon, which indicated the insertion of P*_flgB_-gent* (Figure 6 A and B). Moreover, a primer set within *brpA* demonstrated the displacement of the gene in the mutants (Figure 6 C), while amplification with the *flaB* primers indicated similar amounts of DNA were used for PCR (Figure 6 D). Immunoblot analysis of mutant spirochetes confirmed that the protein was absent from the bacteria grown at 22°C compared to wild type *B. turicatae* grown at that temperature (Figure 7A and B).

### *ΔbrpA* mutants are transmissible by *O. turicata* tick*s*

**Figure 8:**
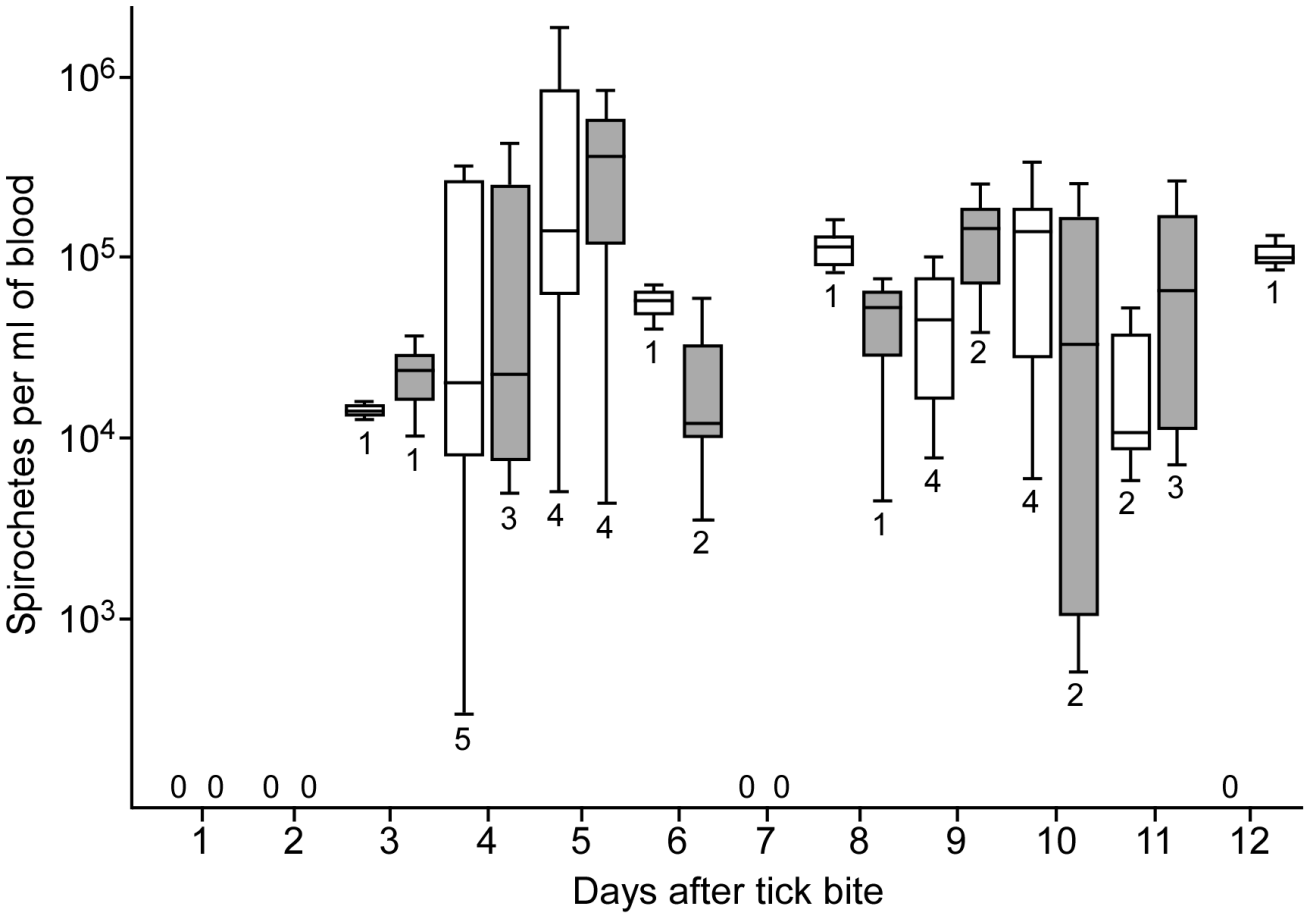
Spirochete densities within murine blood after feeding ticks infected with *ΔbrpA* mutants or wild type spirochetes. The white (mutant infected mice) and grey (wild type infected mice) shaded box and whisker plots represent the lower and upper quartiles, median, and lower and upper extremes of spirochete densities on a given day. The number below each plot and the zero values above the x-axis represents the number of animals infected with mutant or wild type spirochetes on the given day.

Cohorts of uninfected *O. turicata* were fed to repletion on mice infected by needle inoculated with wild type or mutant spirochetes, and salivary gland colonization was confirmed on a portion of ticks by IF-LSCM (data not shown). Ticks infected with wild type or mutant spirochetes were fed on naïve mice and transmission was detected by microscopy and qPCR in two animals within three days (Figure 8). All animals were infected by the fourth day after tick bite, followed by a day of quiescence, and spirochetes repopulated the blood on the eight day. Both wild type and *ΔbrpA* mutant spirochetes attained similar densities within the blood. One animal infected with wild type spirochetes was euthanized before the completion of the experiment, for causes unrelated to this study. Similar results were obtained when transmission experiments were repeated with cohorts of ticks that had molted, which indicated that the mutant spirochetes were maintained transstadially (data not shown). Overall, these results demonstrated that *brpA* was dispensable for tick colonization and establishing infection in the murine model we used. Given the absence of a phenotype in the mutant, complementation was not performed.

## Discussion

The genomes of *Borrelia* spp. display differential gene expression throughout the tick-mammalian infectious cycle [22]–[28]. However, the genes up-regulated by *B. turicatae* during vector colonization are largely unknown. We report the identification of a plasmid localized gene, *brpA*, which was up-regulated during spirochete propagation at 22°C and within *O. turicata* salivary glands. In the *B. hermsii-Ornithodoros hermsi* model of relapsing fever borreliosis, differential production of the variable tick protein (Vtp) and the arthropod-associated lipoprotein (Alp) was demonstrated during vector colonization [22], [28]. While nearly all *B. hermsii* produced Vtp and Alp, BrpA was produced by a subset of spirochetes within the salivary glands of *O. turicata*, further demonstrating the complexity of spirochete gene expression within the vector.

With the surface proteome of spirochetes implicated in host adaptation [29]–[33], determining the requirement of identified surface proteins during the tick-mammalian infectious cycle is important toward deciphering pathogenic mechanisms. Expanding on work accomplished in *Borrelia burgdorferi*, Battisti and colleagues first demonstrated the feasibility of site-specific mutagenesis by electro-transformation in *B. hermsii* [8], [34]. Interestingly, in contrast to *B. burgdorferi*, *B. hermsii* and *B. turicatae* retain their plasmids and infectivity after continuous passage *in vitro* [15], [35], [36]. The genetic stability during cultivation aided us to develop a genetic system to inactivate and evaluate the necessity of the gene product of *brpA* within the tick and mammal.

Deleting *brpA* demonstrated that the mutant spirochetes infected mice by needle inoculation, colonized *O. turicata*, and reached sufficient densities within the salivary glands to ensure subsequent transmission. Our results also indicated that *brpA* was not required for transstadial transmission. With a 30–100% rate of vertical transmission of *B. turicatae* [37], future studies will assess BrpA production within infected eggs and transovarial transmission of mutant spirochetes.

Our findings question the necessity of *B. turicatae* to tightly regulate and retain *brpA*. The gene was dispensable for vector colonization and transmission to mice, and the protein was only detected within a subpopulation of spirochetes. These findings suggest that *B. turicatae* may possess redundant mechanisms, and BrpA may have been replaced with a functionally similar protein. Alternatively, the ecology and maintenance of *B. turicatae* in nature is complex, and a given population of spirochetes within the vector may be preadapted to infect a specific vertebrate host. For example, *B. turicatae* isolates have been acquired from infected domestic dogs in Texas and Florida [6], [38], implicating wild canids to the susceptibility and possible role in the maintenance of the spirochetes. Also, *O. turicata* have been obtained from caves in Texas and gopher tortoise burrows in Florida [39]–[41], suggesting the role of bats and reptiles in the enzootic cycle, respectively. With the immunological and physiological differences between vertebrates, specific phenotypes within a population of spirochetes may have a selective advantage in a given host. The animal model we used to study the role of BrpA in *B. turicatae* was the Swiss Webster mouse, yet *brpA* may be necessary during the bacteria's enzootic cycle, and the experimental murine model may be limiting. As additional animal models are established and with the developed genetic system for *B. turicatae*, a better understanding of the genes required for spirochete maintenance will hopefully be achieved.

## Supporting Information

Figure S1 Detection of *flaB* transcript in *B. turicatae* grown at 22°C and 35°C (A), and evaluation of C_T_ values of *flaB* and *brpA* using genomic DNA (B). When RNA was used as the template (A), squared boxes and diamonds represent *flaB* C_T_ values for spirochetes grown at 22°C and 35°C, respectively. When DNA was used as the template (B), squared boxes and diamonds represent *flaB* and *brpA* C_T_ values, respectively.
(TIF)
