# Supplementary figures and images for: Development of Genetic System to Inactivate a Borrelia turicatae Surface Protein Selectively Produced within the Salivary Glands of the Arthropod Vector
Source: PLoS Negl Trop Dis. 2013 Oct 31;7(10):e2514. doi: 10.1371/journal.pntd.0002514 (PMC3814808; doi:10.1371/journal.pntd.0002514)

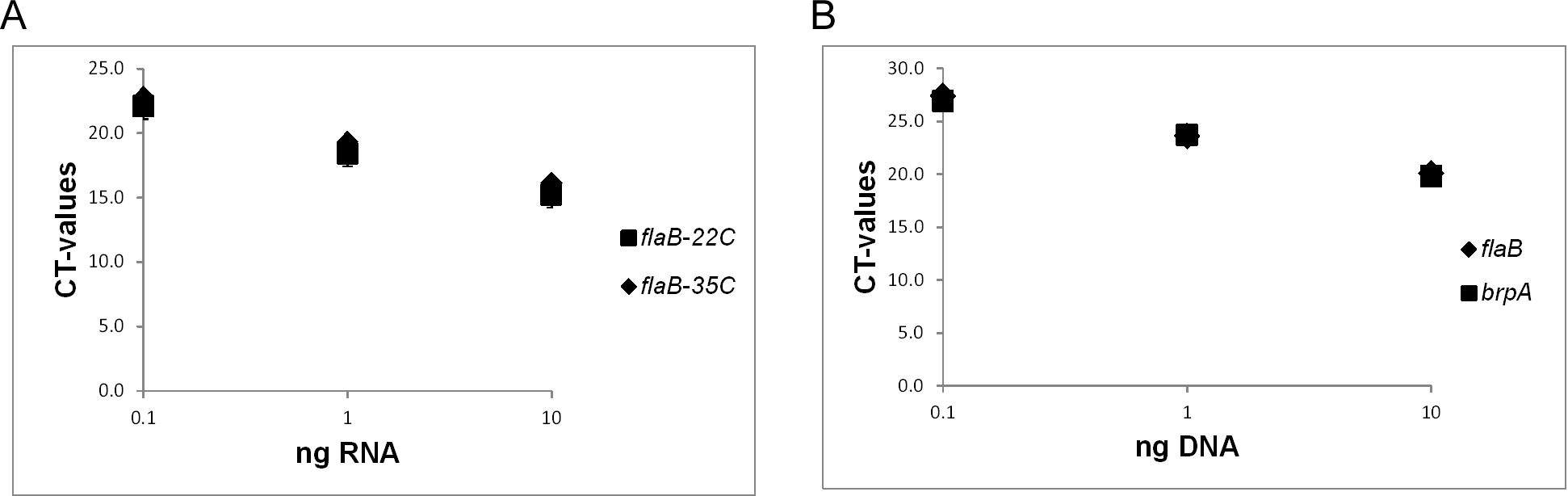

Supplement: Figure S1 — Detection of flaB transcript in B. turicatae grown at 22°C and 35°C (A), and evaluation of CT values of flaB and brpA using genomic DNA (B). When RNA was used as the template (A), squared boxes and diamonds represent flaB CT values for spirochetes grown at 22°C and 35°C, respectively. When DNA was used as the template (B), squared boxes and diamonds represent flaB and brpA CT values, respectively. (TIF) [file pntd.0002514.s001.tif]
